# Hmga2 is necessary for Otx2-dependent exit of embryonic stem cells from the pluripotent ground state

**DOI:** 10.1186/s12915-016-0246-5

**Published:** 2016-03-31

**Authors:** Angelica Navarra, Anna Musto, Anna Gargiulo, Giuseppe Petrosino, Giovanna Maria Pierantoni, Alfredo Fusco, Tommaso Russo, Silvia Parisi

**Affiliations:** Department of Molecular Medicine and Medical Biotechnology, University of Napoli Federico II, via S. Pansini 5, 80131 Naples, Italy; CEINGE, 80145 Naples, Italy; Stazione Zoologica Anton Dohrn, 80121 Naples, Italy; IEOS CNR, 80131 Naples, Italy

**Keywords:** Embryonic stem cell differentiation, Enhancer activation, High-mobility group protein

## Abstract

**Background:**

A crucial event in the differentiation of mouse embryonic stem cells (ESCs) is the exit from the pluripotent ground state that leads to the acquisition of the ‘primed’ pluripotent phenotype, characteristic of the epiblast-like stem cells (EpiLCs). The transcription factors Oct4 and Otx2 play a key role in this phenomenon. In particular, Otx2 pioneers and activates new enhancers, which are silent in ESCs and which control the transcription of genes responsible for the acquisition of the EpiLC phenotype. An important point that remains to be addressed is the mechanism through which Otx2 engages the new enhancers and stably associates with them.

Hmga2 is a member of the high-mobility group family of proteins, non-histone components of chromatin whose expression is high during embryogenesis and becomes low or undetectable in adults. Its high expression during embryogenesis suggests that Hmga2 fulfills important roles in development.

**Results:**

Here, we demonstrate that Hmga2 accumulates soon after the induction of ESC differentiation. Its suppression hampers the exit of ESCs from the pluripotent ground state and their differentiation into EpiLCs. Mechanistically, Hmga2 controls the differentiation process by cooperating with Otx2 in the pioneering of new enhancers. In Hmga2 null induced pluripotent stem cells we observe that Otx2 fails to regulate its target genes upon the induction of differentiation. Hmga2 associates to Otx2-bound loci in EpiLCs, and in Hmga2 KO cells Otx2 is unable to engage and activate the new enhancers, thus indicating that Hmga2 is required for the binding of Otx2 to its cis-elements. We find that this mechanism also operates on the *Hmga2* gene, which is one of the targets of Otx2, thus indicating the existence of a positive feedback loop.

**Conclusions:**

Our findings reveal a novel mechanism necessary for the exit of ESCs from the pluripotent ground state. Upon the induction of ESC differentiation, Otx2 alone or in combination with Oct4 engages new enhancers, which are silent in undifferentiated ESCs. The *Hmga2* gene is activated by Otx2 and Hmga2 protein binds to the enhancers targeted by Otx2, thus facilitating the engagement and/or the stable association of Otx2. Therefore, our results demonstrate that Hmga2 is a key element of the regulatory network that governs the exit of ESCs from the pluripotent ground state.

**Electronic supplementary material:**

The online version of this article (doi:10.1186/s12915-016-0246-5) contains supplementary material, which is available to authorized users.

## Background

Embryonic stem cells (ESCs) are able to self-renew and differentiate in vitro, giving rise to all the cell types of the embryo, thus mimicking the events that take place in vivo during the early stages of development. While enormous progress has been made in understanding the mechanisms that sustain ESC pluripotency [1], the processes governing the exit of ESCs from the pluripotent state and the early steps of differentiation are still not well understood. It is well established that activation of the ERK and GSK3β pathways is necessary for lineage commitment of ESCs [2], and that MAPK phosphatases have an important role in the downregulation of ERK signaling [3]. Recent results indicate that redistribution of βHLH transcription factor (TF) Tfe3 from the nucleus to the cytoplasm, driven by folliculin/Fnip1 and Tsc2, is necessary for the exit from the pluripotent state [4]. Another βHLH TF, Tcf15, primes ESCs for differentiation by downregulating *Nanog* and inducing *Otx2* [5]. Of particular interest is the role of Otx2. This TF, alone and in combination with Oct4, regulates a complex array of genes that marks the very early steps of exit from pluripotency and the acquisition of early post-implantation epiblast cell state (EpiLC) [6–8]. Induction of Otx2 expression and its engagement to genomic loci occur very early during the exit of mouse ESCs from the ground state of pluripotency, indicating that Otx2 acts as a pioneer to engage and activate silent enhancers. However, while accumulation of Otx2 clearly contributes to its own activity, how Otx2 engages new enhancers and stably associates with them remains unclear.

The high-mobility group (HMG) family of proteins form an abundant, heterogeneous, non-histone component of chromatin. Hmga members of this family are highly expressed during embryogenesis and their expression becomes more restricted as fetal development progresses, with low or undetectable expression in adults [9, 10], and becomes abundant in malignant cells in vitro and in vivo, where they have been extensively studied [11]. The high expression of Hmga proteins during embryogenesis suggests that they fulfill important roles in development. Indeed, it has been recently reported that the *Hmga1/Hmga2* double knockout (KO) mice showed high embryonic lethality [12].

Hmga proteins lack transcriptional activity per se, but act by orchestrating the assembly of transcription factor complexes also known as enhanceosomes [13]. They preferentially bind to A/T-rich sequences in close proximity to, or overlapping with, the binding sites of sequence-specific TFs and favor the formation of multi-subunit protein-DNA complexes by modifying the chromatin structure in an ATP-independent fashion [13]. One example of the function of Hmga proteins is that of the *IFN-β* gene promoter. Upon viral infection, the transcription of the *IFN-β* gene depends on the recruitment of several TFs, including NFkB, to an enhancer region positioned in the *IFN-β* gene promoter. The assembly of this complex is dependent on the interaction of Hmga1 with an A/T-rich sequence present in the promoter [14]. Another well-studied example of the role of Hmga in enhanceosome formation is that of the *IL-2Rα* gene. In this case, Hmga1 is upregulated upon stimulation of T cells and binds to A/T-rich sequences in the *IL-2Rα* gene promoter. This recruitment induces a chromatin remodeling that allows several TFs, like Elf-1, STAT5 and others, to interact with their cognate cis-elements, which become accessible only as a consequence of Hmga1-DNA interaction [15]. In the case of the *IL-2Rα* gene promoter, and also in the case of the *α-B-crystallin* gene [16], Hmga proteins interact with A/T-rich sequences positioned on the surface of, or close to, positioned nucleosomes which hamper the binding of sequence-specific TFs. One of the effects of Hmga seems to be that of removing the nucleosomal constraints that prevent the formation of the TF-DNA complexes [13].

Here, we show that Hmga2 accumulates upon the induction of mouse ESC differentiation and its suppression interferes with the transition of ESCs into the epiblast-like state. *Hmga2* gene transcription is regulated by Otx2 and accumulating Hmga2 cooperates with Otx2 in the pioneering activity that allows the switching on of new enhancers that regulate change in the gene expression program that ultimately governs the exit from the pluripotent ground state of ESCs.

## Results

### Hmga2 suppression interferes with ESC differentiation

**Fig. 1:**
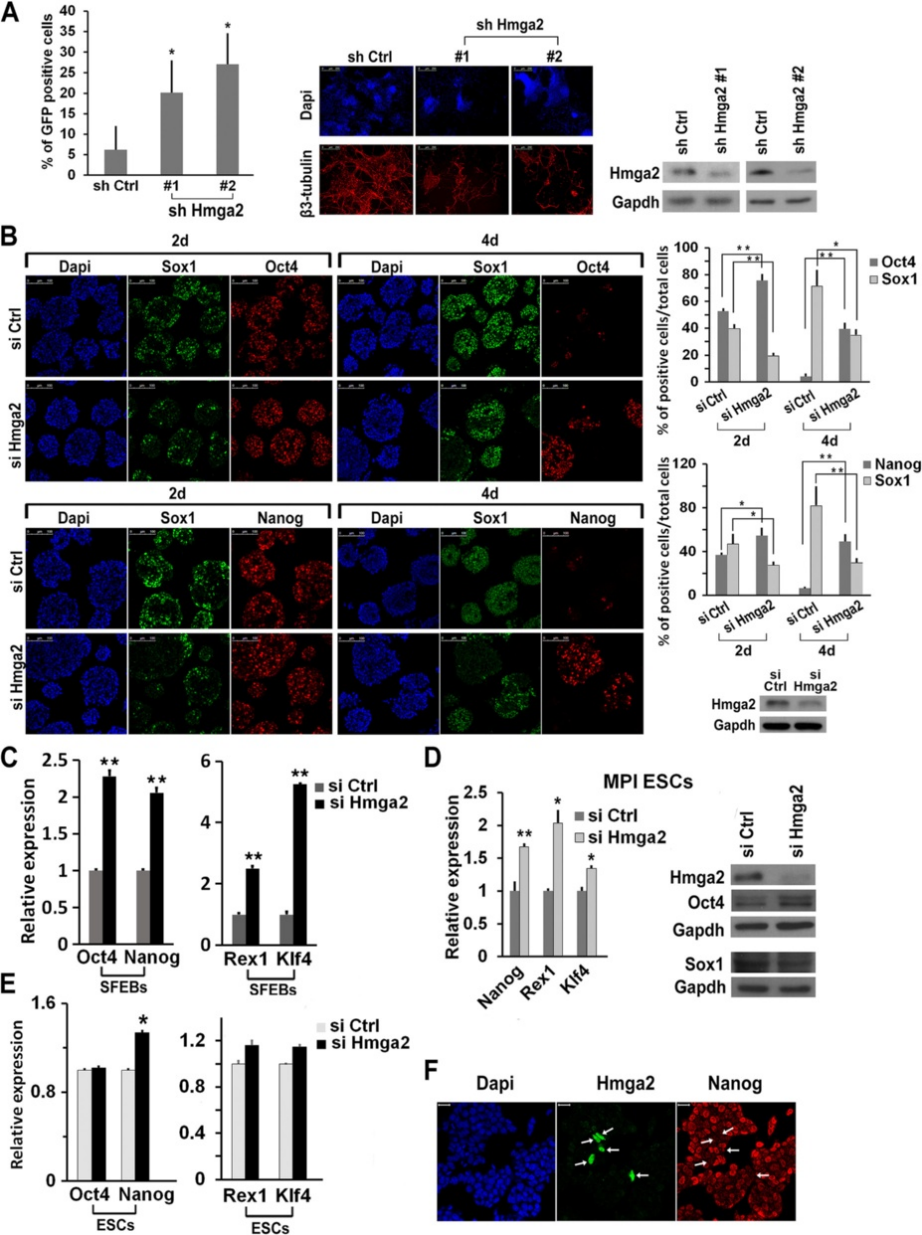
Hmga2 suppression interferes with embryonic stem cell (ESC) differentiation. **a** ESCs expressing GFP under the control of *Nanog* gene promoter were transfected with the control shRNA (sh Ctrl) and two different shRNAs targeting Hmga2, which induced a significant decrease of the Hmga2 protein. Knockdown cells were induced to differentiate into neurons. After 8 days of differentiation, cells were harvested and the number of GFP expressing cells was measured by FACS, subtracting the fluorescent background of parental differentiated E14Tg2a ESCs. The histogram shows the percentage of GFP-positive cells in the indicated samples as mean ± SEM (n = 6). *P <0.05. The efficiency of neuron formation was evaluated at 10 days of differentiation by staining the cells with a neuro-specific antibody (anti-β3-tubulin). Scale bars: 250 μm. **b** Analysis of the phenotype induced by Hmga2 suppression during neuroectodermal differentiation of ESCs. ESCs were transfected with the indicated siRNA, and induced to differentiate through serum-free embryoid body (SFEB) formation for 2 and 4 days. Sectioned SFEBs were then subjected to immunostaining to analyze the presence of stemness (Oct4, Nanog) and neuroectodermal (Sox1) markers. Scale bars: 100 μm. The histograms represent the percentage of Oct4-, Nanog-, and Sox1-positive cells counted in more than six independent fields. *P <0.05, **P <0.01. The efficiency of Hmga2 silencing by siRNA was measured by western blot. **c** qPCR analysis of the expression changes of Oct4, Nanog, Rex1, and Klf4 in differentiated ESCs upon suppression of Hmga2. Data represent mean of three independent experiments (n = 3) ± SEM. *P <0.05. **P <0.01. **d** MPI ESCs were transfected with a control siRNA or a specific Hmga2 siRNA and induced to differentiate into SFEBs. At 4 days of differentiation, SFEBs were collected and the expression of the indicated markers was analyzed by qPCR and western blot. Data represent mean of three independent experiments (n = 3) ± SEM. *P <0.05. **e** qPCR analysis of the expression of Oct4, Nanog, Rex1, and Klf4 in undifferentiated ESCs upon suppression of Hmga2. Data represent mean of independent experiments (Oct4, Rex1, Klf4, n = 3; Nanog, n = 4) ± SEM. *P <0.05. **f** Representative images of the double staining of undifferentiated ESCs with Nanog (*red*) and Hmga2 (*green*) antibodies. The arrows indicate cells expressing Hmga2, which corresponds to cells expressing the lowest level of Nanog. Scale bars: 20 μm

The screening of 20,850 shRNAs interfering with 11,250 independent transcripts, aimed at searching for factors involved in the differentiation of ESCs [17], allowed us to identify, among numerous factors that hamper ESC differentiation, the *Hmga2* gene. Using an ESC line in which GFP is under the control of the *Nanog* gene promoter, we found that, at 8 days after the induction of differentiation, the number of undifferentiated cells, thus still expressing GFP, was significantly higher in Hmga2 knockdown (KD) cells than in control shRNA-transfected cells, while the number of differentiated β3-tubulin positive cells was decreased (Fig. 1a). To measure the effects of Hmga2 suppression, we induced the differentiation of the E14Tg2a ESC line through the formation of serum-free embryoid bodies (SFEBs). As shown in Fig. 1b, wildtype (wt) SFEBs express differentiation-specific markers, like the neural precursor marker Sox1, starting around day 2 of differentiation, while the inner part of the SFEBs contains small groups of cells that express stemness markers like Oct4 and Nanog. The silencing of Hmga2 with siRNAs caused an evident change in the SFEB phenotype, which showed a decreased number of Sox1 positive cells and a great increase of cells that continue to express pluripotency markers (Fig. 1b). According to the results of immunostaining, at 4 days of differentiation, SFEBs derived from *Hmga2* KD cells showed higher levels of Oct4 and Nanog mRNAs than control siRNA-transfected cells (Fig. 1c). Furthermore, Rex1 and Klf4, specific markers of undifferentiated ESCs, which are switched off as soon as ESCs differentiate [18], were still significantly expressed in *Hmga2* KD cells (Fig. 1c). In another mouse ESC line, MPI, the silencing of *Hmga2* affected differentiation, with a phenotype very similar to that observed in the E14Tg2a line: the stemness markers remained elevated up to 4 days after the induction of differentiation, while the neural precursor marker Sox1 failed to accumulate to the levels observed in the cells transfected with the control siRNAs (Fig. 1d).

Hmga2 KD does not significantly modify the expression of stemness markers in undifferentiated cells. However, a slight increase in Nanog expression upon Hmga2 KD was always detected after 24 hours of siRNA transfection (Fig. 1e). Co-immunostaining of wt ESCs with Nanog and Hmga2 antibodies confirmed the wide spectrum of Nanog expression, which varied from very high expression to barely detectable levels, according to the metastable state of undifferentiated ESCs [19]. These experiments showed rare Hmga2 positive cells (less than 1 %) that correspond to cells expressing very low levels of Nanog (Fig. 1f). These results indicate the existence, in undifferentiated ESCs, of a small sub-population of ‘low’ Nanog cells that co-express Hmga2.

All these results indicate that most cells in *Hmga2* KD SFEBs have a phenotype more similar to undifferentiated ESCs, marked by the expression of Oct4, Nanog, Rex1, and Klf4, than to differentiated cells, thus suggesting that Hmga2 KD interferes with the first phase of differentiation, i.e. the transition of ESCs from the pluripotent ground state into the epiblast-like state.

### Hmga2 suppression interferes with the transition of ESCs into EpiLCs

**Fig. 2:**
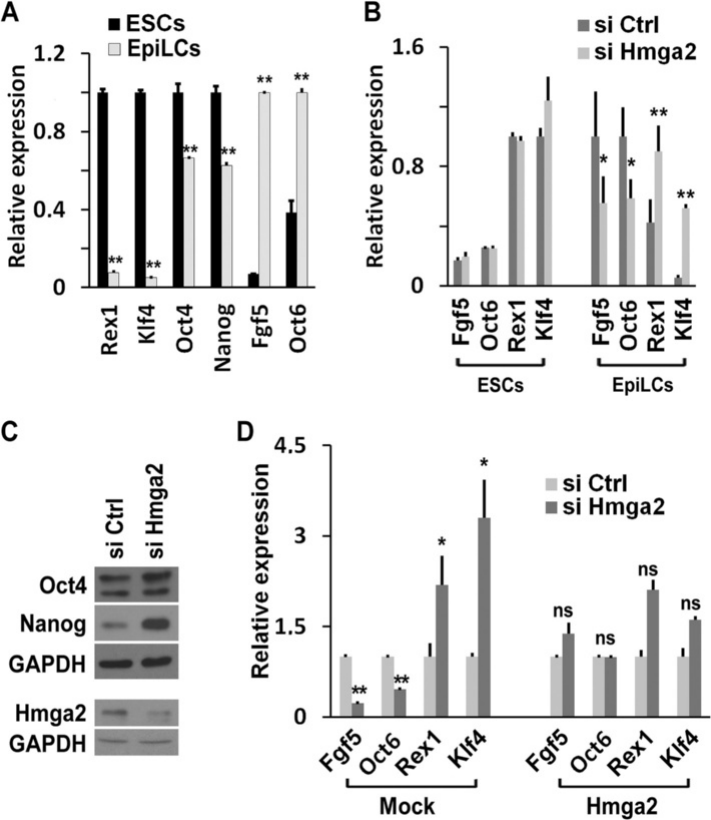
Hmga2 suppression interferes with the transition of embryonic stem cells (ESCs) into epiblast-like stem cells (EpiLCs). **a** qPCR analysis to compare the expression levels of the indicated genes in ESCs versus EpiLCs. Data are reported as means (n = 3) ± SEM of biological replicates. **b** Comparison of the gene expression changes of ESC (Rex1 and Klf4) and EpiLC (Fgf5 and Oct6) markers in ESCs and EpiLCs upon *Hmga2* silencing. Data are reported as means (n = 3) ± SEM of biological replicates (in the case of Fgf5 and Oct6 in EpiLC n = 4). **c** Western blot analysis to measure the expression level of Oct4, Nanog, and Hmga2 in EpiLCs derived from ESCs transfected with the indicated siRNAs. **d** Gene expression changes as a consequence of Hmga2 knockdown in EpiLCs transfected with GFP (mock) or Hmga2 under the control of the β-actin gene promoter. qPCR data are reported as mean of relative mRNA expression ± SEM of biological replicates (Fgf5 and Rex1, n = 4; Oct6 and Klf4, n = 3). *P <0.05, **P <0.01, ns: not significant

To explore this possibility in a more homogenous system, we induced ESC differentiation in culture conditions that stabilize the EpiLC state [20]. As expected, around day 2 after the induction of the differentiation in wt cells, Rex1, Klf4, Oct4, and Nanog decreased, and Fgf5, Oct6, specific markers of the epiblast, accumulated (Fig. 2a). The silencing of *Hmga2* results in a less pronounced accumulation of Fgf5, Oct6, while Rex1 and Klf4 remained significantly expressed (Fig. 2b). In addition, Oct4 and Nanog levels were higher in Hmga2 KD EpiLCs than in control cells (Fig. 2c). The ectopic expression of the Hmga2 cDNA, insensitive to silencing, rescued the accumulation of the epiblast markers and the downregulation of ESC-specific stemness markers (Fig. 2d). In summary, these results indicate that, following the induction of differentiation, Hmga2 KD cells remain more similar to undifferentiated ESCs than to EpiLCs, thus suggesting that Hmga2 is necessary for the exit of ESCs from the pluripotent ground state.

### Hmga2 null induced pluripotent stem cells (iPSCs) fail to exit from the pluripotent ground state

**Fig. 3:**
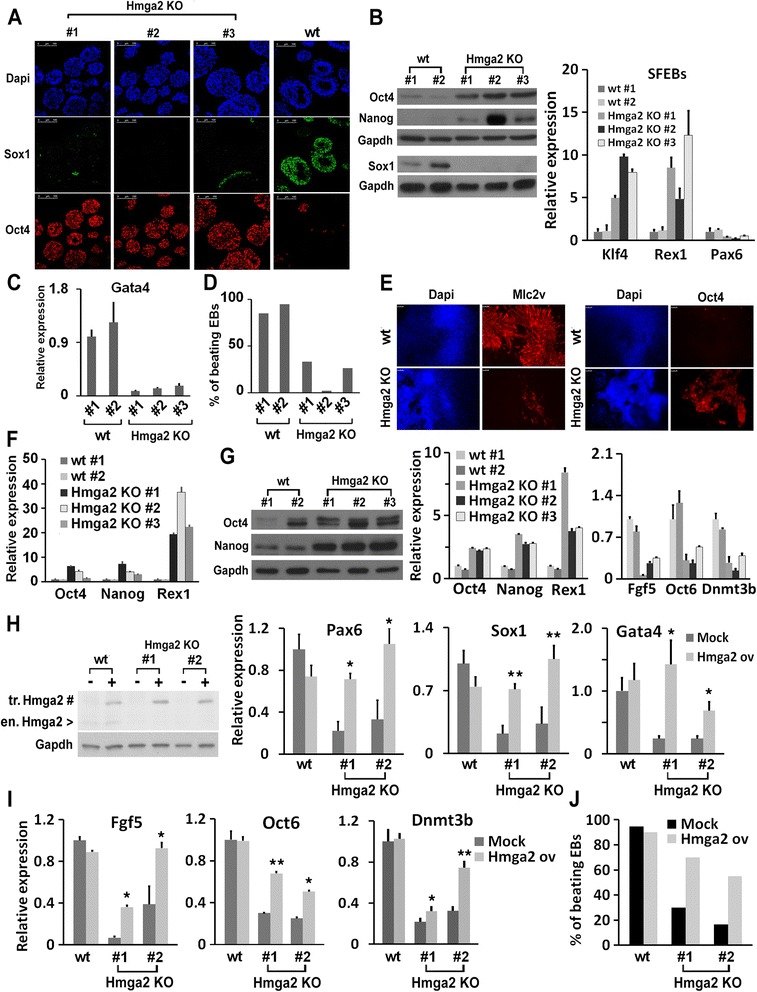
Induced pluripotent stem cells (iPSCs) derived from Hmga2 knockout (KO) embryonic fibroblasts (MEFs) are unable to properly differentiate. **a** iPSC clones derived from wildtype (wt; #1 and #2) and Hmga2 KO (#1, #2, #3) MEFs and grown under 2i + leukemia inhibitory factor (LIF) conditions are induced to differentiate into neuroectoderm through serum-free embryoid body (SFEB) formation for 4 days. Sectioned SFEBs were stained for neuroectodermal (Sox1) and stemness (Oct4) markers. Scale bars: 100 μm. **b** Western blot analysis to evaluate the expression of the indicated proteins in 4 day-differentiated SFEBs derived from different wt and Hmga2 KO iPSC clones and qPCR analysis of the indicated mRNAs. qPCR data are reported as means of relative mRNA expression ± SEM of two biological replicates for each of the indicated wt or Hmga2 KO iPSC clones. **c** The expression of the endodermal marker Gata 4 was evaluated by qPCR in wt and KO Hmga2 iPSCs after 4 days of differentiation as SFEBs. qPCR data represent mean ± SD (n = 2) for each of the indicated wt or Hmga2 KO iPSC clones. **d** Different clones of wt and Hmga2 KO iPSCs were induced to differentiate into cardiomyocytes through the hanging drops method. Histogram reports the percentage of embryoid bodies showing rhythmically contracting areas (beating hearts) on day 13 of differentiation. **e** Immunofluorescence analysis showing the extent of the areas positive for the cardiac ventricular marker myosin Mlc2v or the stemness marker Oct4 in wt and Hmga2 KO iPSCs differentiated for 13 days through the hanging drop method. **f** The expression of the stemness markers was measured by qPCR analysis in wt and Hmga2 KO iPSCs differentiated for 13 days through the hanging drop method. qPCR data represent mean ± SD (n = 2) for each of the indicated wt or Hmga2 KO iPSC clones. **g** The expression of Oct4 and Nanog was analyzed by western blot in wt and Hmga2 KO clones induced to differentiate into EpiLCs. The relative expression of the indicated genes was measured by qPCR analysis in EpiLCs obtained from wt and Hmga2 KO iPSC clones. qPCR data represent means of independent experiments (n = 2; in the case of Oct6 n = 3) ± SD for each of the indicated wt or Hmga2 KO iPSC clones. **h** The relative expression of Pax6, Sox1, and Gata4 was measured by qPCR analysis in SFEBs obtained from wt and Hmga2 KO iPSC clones, stably transfected with an Hmga2 or GFP (Mock) under the control of *β-actin* gene promoter. qPCR data represent the mean of three independent experiments (n = 3) ± SEM for each of the indicated wt or Hmga2 KO iPSC clones. *P <0.05. The expression levels of endogenous Hmga2 (>) and of the transfected form (#) are reported in the western blot. **i** The relative expression of Fgf5 and Oct6 was measured by qPCR analysis in EpiLCs obtained from wt and Hmga2 KO iPSC clones, stably transfected with Hmga2 or GFP (Mock) under the control of *β-actin* gene promoter. qPCR data represent mean ± SEM of three independent experiments (n = 3) for each of the indicated wt or Hmga2 KO iPSC clones. *P <0.05. **j** Wt and Hmga2 KO iPSCs, transfected or not with Hmga2 cDNA, were induced to differentiate into cardiomyocytes through the hanging drops method. The histogram reports the percentage of embryoid bodies showing rhythmically contracting areas (beating hearts) on day 13 of differentiation

To study ESC differentiation in the absence of Hmga2, we generated iPSCs from embryonic fibroblasts (MEFs) of *Hmga2* KO embryos. We found that the absence of Hmga2 resulted in a reduced reprogramming efficiency, as demonstrated by the decreased number of alkaline phosphatase positive colonies compared to iPSCs derived from wt MEFs (Additional file 1: Figure S1A) and by the decreased number of Nanog-positive cells (Additional file 1: Figure S1B). Undifferentiated *Hmga2* KO iPSCs grown under 2i conditions appeared indistinguishable from the wt iPSCs, with a normal pattern of expression of pluripotency markers (Additional file 1: Figure S1C). By using the CFSE proliferation assay, we found that the proliferation rate of *Hmga2* KO iPSCs, grown in undifferentiated or differentiated conditions, was not distinguishable from that of wt iPSCs (Additional file 2: Figure S2A). However, when we induced *Hmga2* KO iPSCs to differentiate as SFEBs by removing the two inhibitors, we observed an almost complete block of differentiation. In contrast to the high number of Sox1-positive cells observed in differentiated wt iPSCs, almost all *Hmga2* KO cells remained positive to Oct4, with only rare cells expressing Sox1 (Fig. 3a). Furthermore, western blot analyses showed that both Oct4 and Nanog are higher in SFEBs from *Hmga2* KO iPSCs than in those derived from wt iPSCs, and confirmed the absence of Sox1 induction (Fig. 3b). Accordingly, mRNA levels of Rex1 and Klf4 remained elevated, while those of Pax6 were not induced (Fig. 3b). This phenotype was consistent among several independent *Hmga2* KO iPSC clones and is maintained at least until 8 days of differentiation, the time required for neuron formation in wt cells (Additional file 2: Figure S2B).

The absence of Hmga2 also impairs the formation of other lineages. Indeed, we found that the appearance of GATA4, a marker of endoderm, is significantly compromised in Hmga2 KO SFEBs (Fig. 3c). Moreover, as reported in Fig. 3d,e, the absence of Hmga2 resulted in an almost complete failure of the expression of cardio-specific myosin and of the generation of beating cardiomyocytes. This phenomenon is again accompanied by a sustained expression of stemness markers (Fig. 3f).

Subsequently, we studied the phenotype of *Hmga2* null iPSCs induced to differentiate into EpiLCs. In these conditions, the Hmga2 KO resulted in an almost complete block of differentiation as demonstrated by the observation that, compared to wt cells, Oct4, Nanog, and Rex1 are still expressed at high levels and the accumulation of Fgf5, Oct6, and Dnmt3b was strongly reduced (Fig. 3g). To address whether Hmga2 re-expression in Hmga2 null iPSCs could rescue the differentiation ability, we generated Hmga2 KO and wt iPSC clones stably transfected with Hmga2 or GFP, as a control. As shown in Fig. 3h,i, the re-expression of Hmga2 rescued the expression of the neuroectodermal differentiation markers Pax6 and Sox1 and the endodermal marker GATA4, when the cells were differentiated as SFEBs, and of the epiblast markers Fgf5, Oct6, and Dnmt3b, when the cells were induced to differentiate into EpiLCs. Hmga2 re-expression in Hmga2 null iPSCs also rescued the ability of the cells to differentiate into cardiomyocytes (Fig. 3j).

Altogether, the results described above demonstrated that Hmga2 is necessary for the transition from ESCs into EpiLCs.

### Otx2 controls *Hmga2* gene expression in EpiLCs

**Fig. 4:**
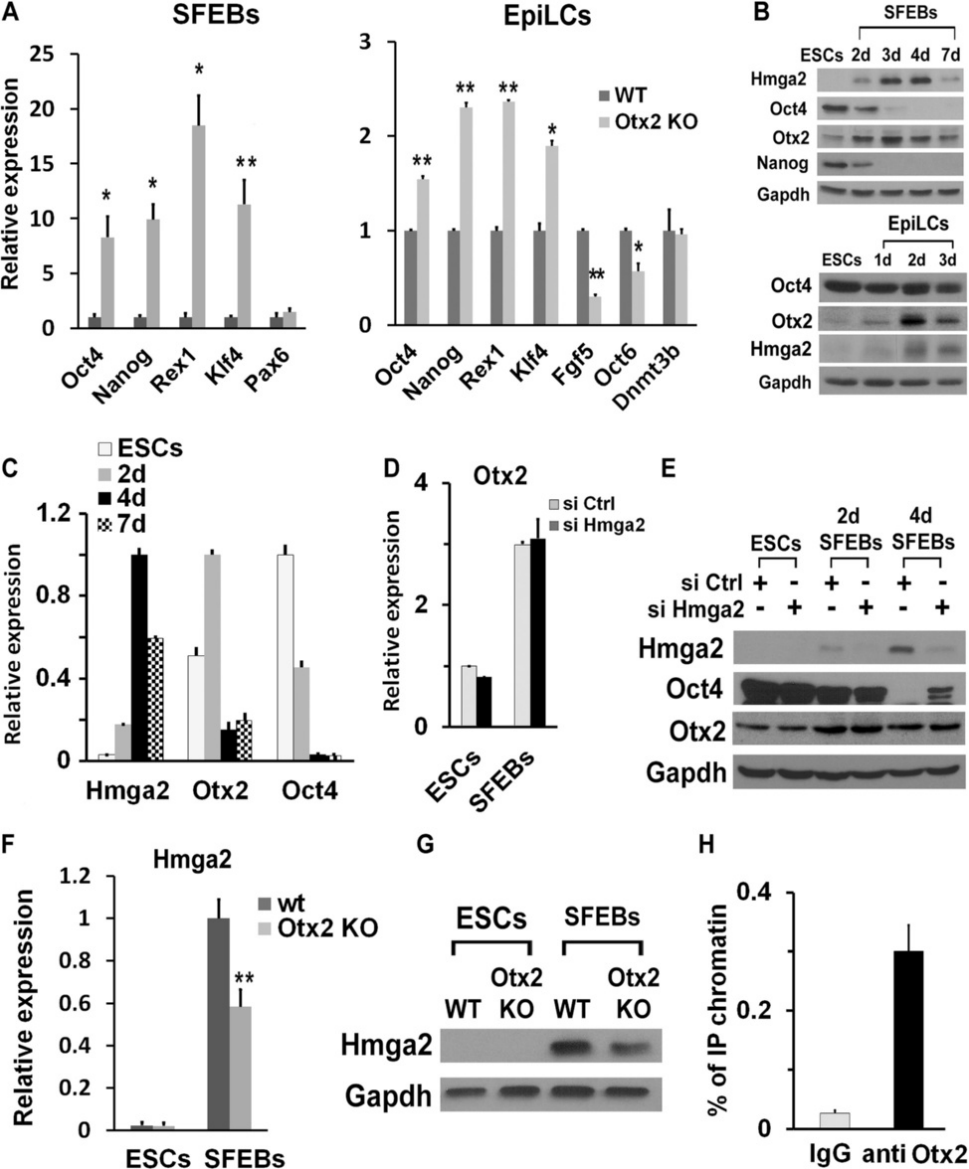
Otx2 controls Hmga2 expression in epiblast-like stem cells (EpiLCs). **a** Phenotypic analysis of Otx2 knockout (KO) and wildtype (wt) differentiated embryonic stem cells (ESCs). The indicated markers were analyzed by qPCR at 4 days of differentiation as serum-free embryoid bodies (SFEBs) or into EpiLCs. qPCR data represent the means of independent biological replicates (Oct4, n = 4; Nanog, Rex1, and Pax6, n = 3; Klf4, n = 5) ± SEM. *P <0.05, **P <0.01. **b** Western blot analysis of undifferentiated and differentiated ESCs. Expression levels of Hmga2, Oct4, and Otx2 were measured in undifferentiated ESCs, during neuroectodermal differentiation through SFEBs and in EpiLCs. **c** Expression profile of Otx2, Oct4, and Hmga2 mRNAs at the indicated time points during ESC differentiation analyzed by qPCR. Data represent mean ± SD from two independent biological replicates (n = 2). **d** Otx2 mRNA levels in undifferentiated ESCs and in SFEBs at day 4 transfected with control or Hmga2 siRNAs. Data represent mean ± SD from two independent biological replicates (n = 2). **e** Oct4 and Otx2 expression in SFEBs at 2 and 4 days of differentiation derived from ESCs transfected with the indicated siRNAs. **f** Wt and Otx2 KO ESCs were induced to differentiate into SFEBs for 4 days. The expression of Hmga2 was analyzed by qPCR. Data represent mean of three independent experiments (n = 3) ± SEM. **P <0.01. **g** Hmga2 protein levels were measured in wt and Otx2 KO undifferentiated ESCs and 4-day differentiated SFEBs. **h** Otx2 association with the genomic region upstream Hmga2. ChIP-qPCR analysis of Otx2 binding to the genomic region upstream the transcriptional start site of Hmga2 (–9 kb) in EpiLCs. qPCR data represent mean of four independent experiments (n = 4) ± SEM

Several recent papers have demonstrated that Otx2 plays a key role in the exit of ESCs from the pluripotent ground state [6–8]. The analysis of the phenotype of Otx2 KO ESCs (Fig. 4a) confirmed that Otx2 is required to allow proper ESC differentiation and in particular the transition from ESCs into EpiLCs [6]. The possibility that these two proteins may functionally interact is further supported by their expression profiles: during SFEB differentiation, Oct4 and Nanog levels rapidly decline, becoming undetectable at day 4, while Otx2 increases at day 2, maintains high levels of expression up to day 4 and, at later time points, decreases at both the protein and mRNA levels (Fig. 4b,c). Hmga2 protein is undetectable by western blot in undifferentiated ESCs, appears soon after induction of differentiation of ESCs into SFEBs (day 2), accumulates up to day 4 (Fig. 4b,c) and subsequently decreases. Similarly, the transition of ESCs into EpiLCs was accompanied by the accumulation of Hmga2 (Fig. 4b). In both cases, the induction of Otx2 slightly precedes that of Hmga2.

The suppression of Hmga2, which strongly affects the gene expression profiles of differentiating ESCs (Figs. 1 and 2), had no effects on the expression profile of Otx2. During differentiation, Otx2 accumulates in Hmga2 KD cells to the same extent as in wt cells (Fig. 4d,e). Conversely, we observed that Hmga2 mRNA and protein were decreased in Otx2 KO SFEBs (Fig. 4f,g). By examining previously published chromatin immunoprecipitation-sequencing (ChIP-seq) data where *Otx2* gene targets were analyzed, we found that *Hmga2* is one of the candidate targets [8]. ChIP experiments in wt EpiLCs allowed us to observe that Otx2 binds to a region upstream of the transcriptional start site in the *Hmga2* gene, confirming the previously published ChIP-seq data (Fig. 4h). Inspection of this DNA sequence demonstrated the presence of a bona fide cis-element for *Otx2* that could be responsible for the regulation of this gene upon differentiation of ESCs. These findings can explain, at least in part, the changes in expression of Hmga2 during the first steps of differentiation.

### Hmga2 assists Otx2 in the pioneering of new enhancers

**Fig. 5:**
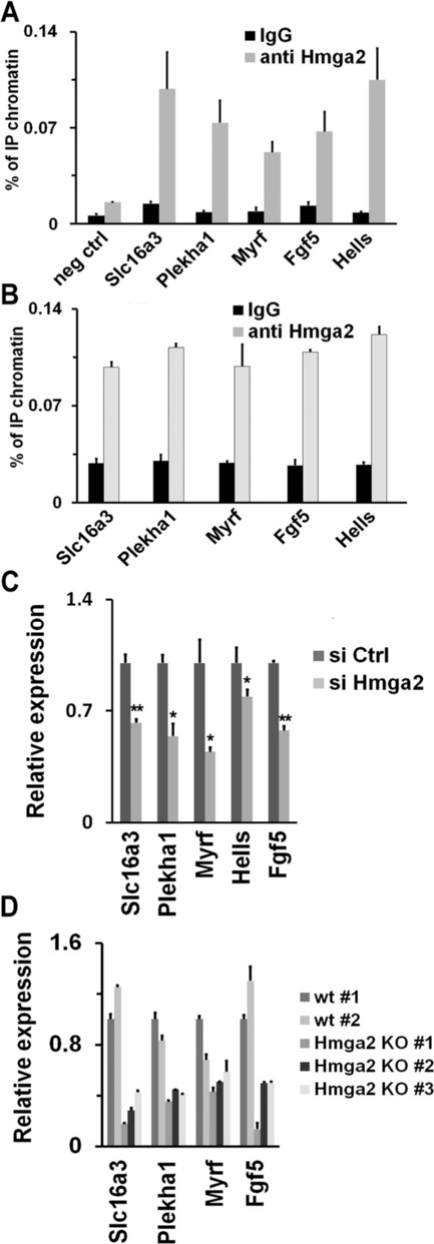
Hmga2 is involved in the regulation of Otx2 target genes upon the exit of embryonic stem cells (ESCs) from the pluripotent ground state. **a** ChIP-qPCR analysis of the association of Hmga2 to gene targets of Otx2 in wildtype (wt) epiblast-like stem cells (EpiLCs). The negative control (neg ctrl) corresponds to the region 10.1 kb upstream of the Hmga2 transcriptional start site. qPCR data represent means of three independent experiments (n = 3) ± SEM. **b** ChIP-qPCR analysis of the association of Hmga2 to gene targets of Otx2 in wt induced pluripotent stem cells (iPSCs) induced to differentiate. qPCR data represent means of two independent experiments (n = 2) ± SD. **c** The relative level of the indicated mRNAs was measured by qPCR in EpiLCs derived from ESCs transfected with si Ctrl and si Hmga2. qPCR data represent mean of three independent experiments (n = 3) ± SEM. *P <0.05, **P <0.01. **d** qPCR analysis of the expression of the indicated genes in wt and Hmga2 KO iPSC clones differentiated into EpiLCs. qPCR data represent means of two independent experiments (n = 2) ± SD for each of the indicated wt or Hmga2 KO iPSC clones

During the transition from ESCs to EpiLCs, many gene promoters are bound by Otx2 alone or by Otx2 and Oct4 [7, 8]. The presence of Otx2 both in ESCs and EpiLCs suggests that Otx2 is likely to require additional factors to access its cognate cis-elements. Of note, one of the best documented functions of HMG proteins is to assist the engagement of transcription factors to inducible gene promoter regions, thus leading to the formation of the so called enhanceosomes. This function requires the association of HMG proteins with the minor grove of A/T-rich DNA sequences, close to or overlapping with the cis-elements targeted by specific TFs. No clear consensus binding sequence has been found in the published ChIP experiments of Hmga2 association to chromatin in a human tumor cell line [21] except for A/T-rich stretches. The Otx2 consensus binding motif contains 4–6 consecutive A = T pairs [8]. Thus, we looked at the possible interaction of Hmga2 with Otx2 binding sites. We first assessed, in stabilized EpiLCs derived from E14Tg2a or wt iPSCs, the association of Hmga2 with several loci included in the published list of Otx2 ChIP-seq targets and we found that Hmga2 was always associated with Otx2 binding sites such as, for example, enhancers of the *Hells*, *Slc16a3*, *Plekha1*, *Fgf5*, and *Myrf* genes (Fig. 5a,b). We then examined whether Hmga2 suppression by RNAi affects the expression of Otx2 targets and found a decreased expression of all the genes analyzed (Fig. 5c). Similarly, in Hmga2 KO iPSCs the expression of Otx2 target genes was greatly decreased (Fig. 5d).

**Fig. 6:**
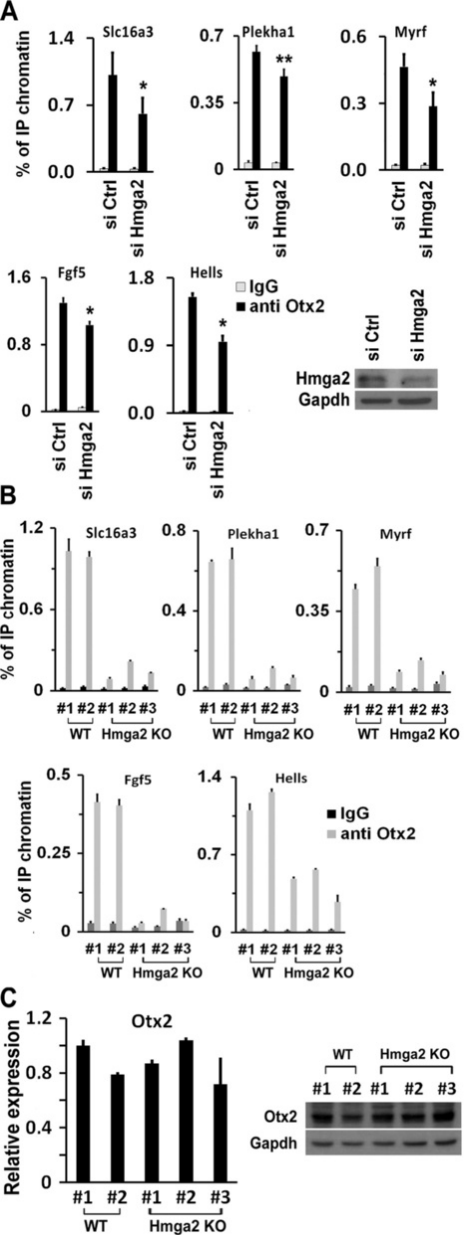
Hmga2 is necessary for the pioneering activity of Otx2 in the engagement of new enhancers during the transition from embryonic stem cells (ESCs) into epiblast-like stem cells (EpiLCs). **a** Otx2 association with the indicated genes upon Hmga2 silencing. ESCs transfected with si Ctrl or si Hmga2 were induced to differentiate into EpiLCs and the Otx2 binding to the enhancers of indicated genes was measured by ChIP-qPCR. Data are presented as mean ± SEM of independent biological replicates (Plekha1, FGF5, Hells, n = 3; Slc16a3, Myrf, n = 4). *P <0.05, **P <0.01. **b** ChIP-qPCR analysis of the binding of Otx2 to the enhancers of the indicated genes in EpiLCs derived from wt and Hmga2 KO induced pluripotent stem cell (iPSC) clones. qPCR data represent mean of two independent experiments (n = 2) ± SD for each of the indicated wt or Hmga2 KO iPSC clones. **c** Otx2 mRNA and protein expression levels in wt and KO iPSCs induced to differentiate into EpiLCs. qPCR data are means of three independent experiments (n = 3) ± SD

These results suggest a close relationship between Otx2-dependent gene regulation and the availability of Hmga2 bound to Otx2 cis-elements. Therefore, we asked whether Hmga2 cooperates with Otx2 by influencing its capacity to engage new enhancers. To address this possibility, we explored whether suppression of Hmga2 affects binding of Otx2 to its cognate loci. ChIP experiments, performed in EpiLCs derived from Hmga2 KD cells, showed that the binding of Otx2 to selected targets is significantly decreased (Fig. 6a). When we tested Hmga2 null cells, the binding of Otx2 to its targets was greatly decreased or almost completely abolished (Fig. 6b and Additional file 3: Figure S3A).

The decreased binding of Otx2 to cognate cis-elements in Hmga2 KD and KO cells did not depend on a lack of induction of Otx2. Indeed, as shown in Fig. 6c, the behavior of Otx2 upon the induction of differentiation was identical in wt and in Hmga2 KO cells. Furthermore, the ability of Otx2 to associate with chromatin in undifferentiated conditions (ESCs) was not modified by the suppression of Hmga2 (Additional file 3: Figure S3B).

**Fig. 7:**
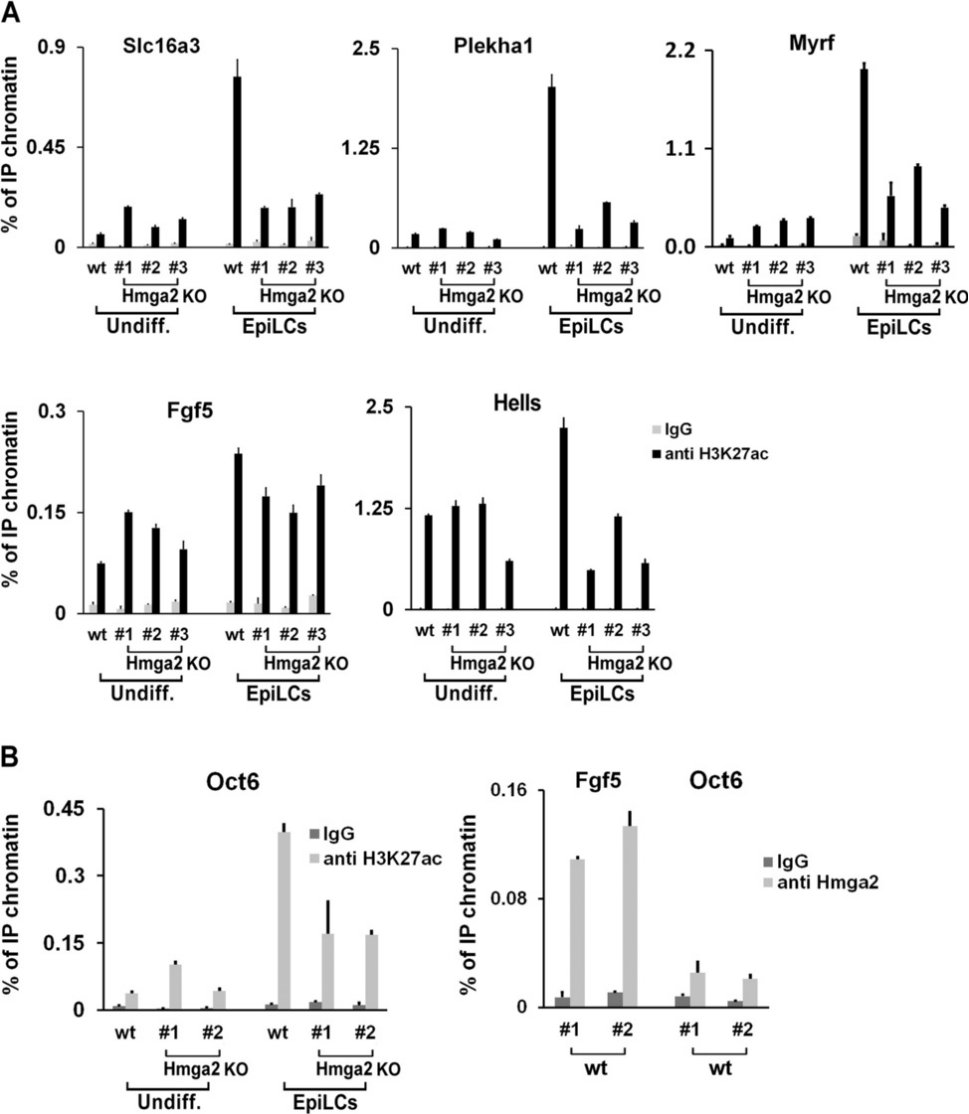
Enhancer activation in wildtype (wt) and Hmga2 knockout (KO) cells. **a** The activation of the indicated enhancers was analyzed by measuring the levels of H3K27ac by ChIP-qPCR in undifferentiated and differentiated wt and Hmga2 KO induced pluripotent stem cells (iPSCs). qPCR data represent means of two independent experiments (n = 2) ± SD for each of the indicated wt or Hmga2 KO iPSC clones. **b** The levels of H3K27ac on Oct6 enhancer were measured by ChIP-qPCR in undifferentiated and differentiated wt and Hmga2 KO iPSCs. The right panel reports the ChIP of Hmga2 that demonstrates the absence of binding to the Oct6 enhancer in EpiLCs. qPCR data represent means of two independent experiments (n = 2) ± SD for two wt iPSC clones

In wt cells, the binding of Otx2 was accompanied by the activation of the enhancers, as demonstrated by the increased acetylation of H3K27, while in Hmga2 KO cells, the decreased binding of Otx2 resulted in decreased levels of H3K27Ac (Fig. 7a). Therefore, the decrease or the absence of Hmga2 reduces the engagement and the activation of new enhancers by Otx2, upon the induction of differentiation. We also explored the effects of Hmga2 KO on an Otx2-independent enhancer, present upstream of the Oct6 gene [7, 8], which is activated upon the exit from the naïve state. As shown in Fig. 7b, the absence of Hmga2 caused a decrease of the Oct6 enhancer activation, as demonstrated by the reduced levels of H3K27Ac marker, although Hmga2 is not associated with this enhancer in wt cells. This phenomenon is likely a consequence of the phenotype caused by the suppression of Hmga2, i.e. a general impairment of the transition into the epiblast-like state.

**Fig. 8:**
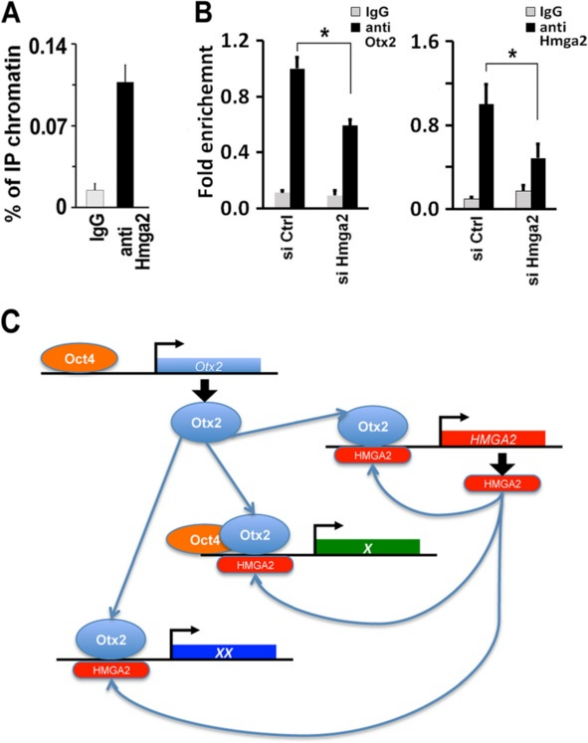
Hmga2 assists Otx2 in the activation of the *Hmga2* gene. **a** The association of Hmga2 to the genomic region upstream of its own gene was measured by ChIP-qPCR in epiblast-like stem cells (EpiLCs). qPCR data represent mean of independent biological replicates (n = 5) ± SEM. **b** Otx2 binding to the genomic region upstream of Hmga2 gene (left panel) was measured by ChIP-qPCR in EpiLCs derived from embryonic stem cells transfected with the indicated siRNAs. Data are presented as means of independent experiments (n = 4) ± SEM. *P <0.05. The right panels shows the Hmga2 binding to its own enhancer in EpiLCs upon suppression of Hmga2. Data are presented as means of independent experiments (n = 3) ± SEM. * *P* <0.05. **c** Model illustrating the cooperation of Otx2 and Hmga2 in transcriptional control during the exit from the pluripotent ground state

Otx2 controls transcription of Hmga2 gene promoter, thus explaining its induction during ESC differentiation (Fig. 4f,h). We examined whether Hmga2 positively acts on its own promoter by enhancing Otx2 binding. By performing ChIP for Hmga2 we found that this protein indeed interacts with the same region, upstream of the transcriptional start site, where Otx2 binds (Fig. 8a). Additionally, we observed that Hmga2 suppression resulted in a decreased binding of Hmga2 itself and Otx2 to its cis-element (Fig. 8b). These results indicate a close regulatory crosstalk between Hmga2 and Otx2.

## Discussion

Differentiation of mouse ESCs implies the exit from the pluripotent ground state and the transition into the epiblast-like state. Here, we show that Hmga2, a chromatin-associated protein, is necessary for the transition of mouse ESCs from the naïve pluripotent state into the epiblast-like state. While several results have indicated a clear role of Hmga2 in hematopoietic [22], stromal [23], and neural [24, 25] stem cell function, and although Hmga2 is expressed at the highest levels in mammalian embryos, the physiological role of this protein in the early steps of development was still unclear [26, 27]. The results of this work assign a precise role to this protein: in Hmga2 KD ESCs and KO iPSCs, the induction of differentiation markers is decreased or completely abolished, while pluripotency markers continue to be expressed. The results we show also demonstrate that the function of Hmga2 concerns with the earliest step of ESC differentiation, i.e. their transition into EpiLCs, because Hmga2 KD and KO prevents the accumulation of Fgf5, a marker of EpiLCs. Mechanistically, we demonstrate that Hmga2 is required for and directly involved in the engagement and activation of new enhancers by Otx2. Recent results have demonstrated that Oct4 and Otx2 are responsible for the regulation of genes that characterize the exit from the pluripotent ground state [7, 8]. Therefore, upon the induction of ESC differentiation, Oct4 activates the transcription of the *Otx2* gene and, in turn, Otx2 accumulates and starts the engagement of new enhancers, including that controlling the transcription of the *Hmga2* gene. Hmga2 favors the pioneering and/or the stable association of Otx2 with the new enhancers, and supports a positive feedback loop that sustains the Otx2-dependent transcription of the *Hmga2* gene itself (Fig. 8c). The precise mechanism through which Hmga2 affects the DNA binding capacity of Otx2, and in turn its pioneering of new enhancers, remains to be addressed. One possibility is that Hmga2 affects DNA and/or the chromatin state, thus favoring the engagement and/or the stable association of Otx2 to its cis-element. Most information on the function of Hmga proteins derives from the study of Hmga1. In several cases, Hmga1 interacts with A/T-rich sequences close to or overlapping with the binding elements of TFs. One example is that of the INF-β gene promoter, where Hmga1 binds to A/T-rich sequences overlapping with the NF-kB and ATF-2 cis-elements, thus favoring the formation of an enhanceosome [14]. These sequences are often located on the surface of, or close to, positioned nucleosomes that hamper the binding of sequence-specific TFs. One of the effects of Hmga1 seems to be that of favoring the removal of nucleosomal constraints that prevent the formation of TF-DNA complexes [15, 16]. The crucial role of Hmga2 in transition from ESCs into EpiLCs is also supported by the observation that, when we generated iPSCs from Hmga2 KO MEFs, the efficiency of the reprogramming was significantly reduced. This observation is in agreement with recent results indicating that Hmga2 overexpression facilitates the efficient conversion of somatic cells into human induced neural stem cells [28]. One possible explanation of these phenomena is that Hmga2 favors the expansion of iPSCs rather than their generation. However, our results clearly show that Hmga2 KO iPSCs have the same proliferative capacity of the normal iPSCs. On the other hand, several results, including those presented in this paper, show that Hmga2 has a crucial role in chromatin remodeling assisting the engagement of new enhancers by some transcription factors. Thus, it cannot be excluded that, according to our observations in ESC differentiation, Hmga2 could also favor the transition from an enhancer occupancy specific of differentiated cells towards that of less differentiated cells, thus favoring reprogramming.

The differentiation defects observed in Hmga2 KO iPSCs appear more pronounced compared to those of Otx2 KO ESCs. Indeed, we have observed that the differentiation of Otx2 KO ESCs into EpiLCs does not lead to an impairment of the epiblast marker Dnmt3b (Fig. 4a). Accordingly, Buecker et al. [7] found that Otx2 KO ESCs grown in 2i + LIF conditions and differentiated into EpiLCs show a minor phenotype with defects in the expression of only certain epiblast-associated genes. Our experiments demonstrated that Hmga2 KO iPSCs grown in 2i + LIF conditions show a more pronounced phenotype when differentiated into EpiLCs, with significant defects of the induction of several epiblast markers (Fgf5, Oct6, and Dnmt3b; Fig. 3g). These differences in the differentiated phenotypes of Otx2 KO ESCs and Hmga2 KO iPSCs suggest that Hmga2 may also have Otx2-independent functions.

## Conclusions

Herein, we demonstrate that the chromatin-associated protein Hmga2 plays a crucial role in the exit of ESCs from the pluripotent ground state. Hmga2 suppression prevents ESC differentiation by interfering with the function of the Otx2 TF. This phenomenon is due to a direct involvement of Hmga2 in the engagement and/or stable association of Otx2 with new enhancers upon the induction of ESC differentiation. Indeed, Hmga2 directly binds to the Otx2 cis-element and, in the absence of Hmga2, Otx2 fails to engage and, thus, activate its target enhancers.

## Methods

### Cell culture, transfection, and differentiation

E14Tg2a (Bay Genomics) mouse ESCs were maintained on feeder-free gelatin-coated plates in the following medium (ESC medium): Glasgow Minimal Essential Medium (GMEM, Sigma) supplemented with 2 mM glutamine (Invitrogen), 100 U/mL penicillin/streptomycin (Invitrogen), 1 mM sodium pyruvate (Invitrogen), 1× non-essential amino acids (Invitrogen), 0.1 mM β-mercaptoethanol (Sigma), 10 % FBS (Hyclone), and 10^3^ U/mL LIF (Euroclone). For transfection, ESCs were plated at 6 × 10^4^ cells/cm^2^ the day before the transfection. Transfections of siRNAs (Invitrogen) and plasmids were performed using Lipofectamine 2000 (Invitrogen) following the manufacturer’s instructions. For neuronal differentiation through monolayer, ESCs were trypsinized into a single cell suspension, collected by centrifugation and resuspended in the following differentiation medium: GMEM supplemented with 10 % Knockout Serum Replacement (both from Invitrogen), 0.1 mM β-mercaptoethanol (Sigma), and 2 mM glutamine (Invitrogen). Then, the cells were plated at low density (3 × 10^3^ cells/cm^2^) on gelatin-coated dishes and differentiation medium was changed on alternated days. ESC differentiation through SFEB formation was obtained by placing 1 × 10^6^ ESCs or iPSCs in 100-mm Petri dishes in differentiation medium. To obtain terminal differentiation into neurons, 4-day differentiated SFEBs were dissociated and plated on polylysine (Sigma) coated plates. The appearance of neurons was still evident at 6 days of differentiation.

The formation of EpiLCs was induced as reported in ref. [7]. In brief, ESCs or iPSCs were dissociated and seeded in fibronectin-coated dishes at a density of 2.5 × 10^5^ cells/cm^2^ in ESC culture conditions. After 18 hours the medium was switched to the following EpiLCs medium: 1 vol of DMEM/F12 combined with 1 vol of Neurobasal medium, supplemented with 0.5 % N2 supplement, 1 % B27 supplement, 1 % KSR, 2 mM glutamine (Invitrogen), 20 ng/mL Activin A (R&D Systems), and 12 ng/mL bFGF (Invitrogen). All the experiments performed in EpiLCs were conducted after 3 days from the switch to EpiLC medium.

MPI ESCs [29] were grown on mitotically inactivated fibroblast feeder layers and maintained in DMEM (Invitrogen) supplemented with 15 % FBS (Hyclone), 0.1 mM β-mercaptoethanol (Sigma), 1 mM sodium pyruvate (Invitrogen), 1× nonessential amino acids (Invitrogen), 2 mM glutamine (Invitrogen), 100 U/mL penicillin/streptomycin (Invitrogen), and 10^3^ U/mL LIF (Chemicon International). The day before transfection, the cells were pre-plated on gelatin-coated plates for 1 hour to remove feeder layer and then on gelatin-coated dishes.

Differentiation into cardiomyocytes was obtained by inducing embryoid body formation through the hanging drops method, essentially as previously described [30, 31]. Briefly, ESCs were trypsinized and resuspended at 8 × 10^4^ cells/mL in the following medium: GMEM supplemented with 2 mM glutamine, 100 U/mL penicillin/streptomycin, 1 mM sodium pyruvate, 1× non-essential amino acids, 0.1 mM β-mercaptoethanol, and 15 % FBS (selected batch). The cell suspension was used to deposit 20-μL drops. After 2 days of incubation, the embryoid bodies formed within the drops were collected and plated in suspension into Petri dishes for 3 days. Then, the embryoid bodies were plated separately onto gelatin-coated 48-well plates for morphological analysis or onto 100-mm tissue culture plates for qPCR.

The Nanog-GFP cell line was generated by transfecting E14Tg2a ESCs with a vector bearing the Nanog minimal promoter (from –322 to +50) upstream to the EGFP reporter [32] and a neomycin selection cassette. The undifferentiated ESCs that had stably incorporated the plasmid were positive for GFP, but became negative when differentiation was induced. Nanog-GFP cells were induced to differentiate into neurons as indicated above. All the cell lines used were mycoplasma free.

### iPSC generation and culture

iPSCs were obtained adapting the methods of Takahashi and Yamanaka [33], as follows. Mouse pMXs-based retroviral vectors for Oct4, Sox2, and Klf4 (Addgene) were transfected into Plat-E cells by using Lipofectamine 2000 (Invitrogen) following the manufacturer’s instructions; 24 hours after transfection, the medium was replaced. Virus-containing supernatant was mixed at a ratio of 1:1:1 in the presence of polybrene (8 μg/mL), and wt and HMGA2 KO MEFs were infected twice with TF transduced virus mix. After 48 hours, the medium was replaced with ESC medium containing 15 % FBS (Hyclone). The medium was changed every 2 days. After 21 days of reprogramming, iPSC clones were isolated by picking and expanded in ESC medium in the presence of 2i (MEK and GSK3 inhibitors; 3 μΜ PD0325901 and 1 μΜ CHIR-99021 (Selleckchem), respectively). The isolated clones were characterized for the expression of stemness markers and for the absence of differentiated markers. The Hmga2 KO iPSCs are available upon request.

### RNA extraction, retro-transcription and real time PCR

Total RNA was extracted by using TRI-Reagent (Sigma). The first-strand cDNA was synthesized according to the manufacturer’s instructions (M-MLV RT kit; New England Biolabs). Real Time RT-PCR was carried out with QuantStudio 7 Flex (Applied Biosystems) using Fast SYBR Green PCR Master mix (Applied Biosystems). The housekeeping GAPDH mRNA was used as an internal standard for normalization, using the 2^-∆Ct^ method. Gene-specific primers used for amplification are listed in Additional file 4.

### Chromatin immunoprecipitation (ChIP)-qPCR analysis

ChIP assays were performed from 2 × 10^6^ ESCs, differentiating EpiLCs, SFEBs, and wt and HMGA2 KO iPSCs for each experiment. Briefly, cells were cross-linked with 1 % formaldehyde for 10 min at room temperature and then the reaction was quenched by adding glycine at a final concentration of 125 mM. The chromatin was then sonicated to an average DNA fragment length of 500–1000 bp. Soluble chromatin extracts of the following antibodies were immunoprecipitated: anti-Oct3/4 (Santa Cruz Biotechnology, #sc-5279), anti-Otx2 (Millipore, #AB9566, 1:125), anti-HMGA2 (Cell Signaling, #5269, 1:100) and anti-H3K27ac (Abcam, #AB4729, 1:300) antibodies. Appropriate IgGs (Abcam, #ab46540, 1:200) were used as a negative control. Supernatant obtained without antibody was used as an input control. After qPCR, the amount of precipitated DNA was calculated relatively to the total input chromatin and expressed as a percentage of total chromatin. The primers used are listed in Additional file 4.

### Plasmid construction

The wt Hmga2 cDNA was obtained by PCR amplification from pCEFL-HA expression vector and subcloned into pCAG-Flag vector by using BamHI and HindIII restriction sites. The mutant form of Hmga2 cDNA insensitive to siRNA was generated by a double round of PCR. Next, cDNA was cloned upstream by FLAG-tag and downstream by the chicken β-actin promoter in the pCAG-FLAG vector by using BamHI and HindIII restriction sites. The oligonucleotides used were:

- Hmga2-BamHI-f: 5′- GGTGGATCCGCCGCCATGAGCGCACGCGGTGAGG-3′
- Hmga2-HindIII-r: 5′- GTCAAGCTTATCCTCCTCTGCGGACTCTTGCG-3′
- Hmga2 mut-f: 5′-GGCCAAGAGGAAGGCCCAGAAAGTGGCCGCCCCCTGTCGTTCAG-3′
- Hmga2 mut-r: 5′- CTGAACGACAGGGGGCGGCCACTTTCTGGGCCTTCCTCTTGGCC-3′

### Antibodies and western blot analysis

Undifferentiated and differentiated ESCs were lysed in a buffer containing 1 mM EDTA, 50 mM Tris-HCl (pH 7.5), 70 mM NaCl, 1 % Triton, and protease inhibitor cocktail (Sigma), and analyzed by western blot. The following primary antibodies were used: mouse Oct3/4 (1:1000 Santa Cruz Biotechnology, #sc-5279), rabbit Nanog (1:1000 Calbiochem-EMD Biosciences, #SC1000), mouse GAPDH (1:1000 Santa Cruz Biotechnology, #sc-32233), goat Sox1 (1:100 Santa Cruz Biotechnology, #sc-17318), rabbit Otx2 (1:500 Abcam, #ab114138), rabbit HMGA2 (1:500 Cell Signaling, #5269), and rabbit HMGA2 (1:500). GAPDH was used as an internal control. Antibody protein complexes were detected by HRP-conjugated antibodies (Anti-rabbit IgG, 1:10000, Amersham Pharmacia, #NA934V; Anti-mouse IgG, 1:5000, Amersham Pharmacia, #NA931V; Anti-goat IgG, 1:20000, Sigma-Aldrich, #A5420) and ECL (Amersham Pharmacia).

### Immunofluorescence

Undifferentiated or monolayer differentiated ESCs were fixed in 4 % paraformaldehyde and permeabilized with 0.2 % TX-100 in 10 % FBS/1 % BSA in 1× PBS for 15 min at room temperature. Thus, the samples were incubated with primary antibodies. Following primary antibody incubation, cells were incubated with appropriate secondary antibodies.

SFEBs were washed once with PBS 1× and fixed in 4 % paraformaldehyde over night at 4 °C with gentle rotation. Dehydration was performed over the following 2 days with increasing percentages of EtOH. On the fourth day, the samples were washed once in Toluene for 40 minutes at room temperature and embedded in paraffin blocks. For immunofluorescence, the slides were washed twice in xylene at room temperature for 3 minutes and then rehydrated with a series of washes in EtOH at decreasing percentage. Then, permeabilization was performed with 0.2 % TX-100 for 5 minutes followed by 2 washes in 1× PBS for 2 minutes, followed by unmasking in citrate buffer once. Primary antibodies were incubated in 10 % FBS/1 % BSA/0.1 % Tween 20/1× PBS for 2–3 hours at room temperature followed by washing and secondary antibody hybridization. Nuclei were counterstained with Dapi (1:5000, Calbiochem, #268298) or DRQ5 (1:1000, Invitrogen, #4084S). The following primary antibodies were used: anti-Oct3/4 (1:200, Santa Cruz, #sc-5279), anti-βIII-tubulin (1:400, Sigma-Aldrich, #T8660) and anti-Sox1 (1:100, Santa Cruz, #sc-17318). The secondary antibodies used were: anti-mouse Alexa 594 and anti-goat Alexa 488 (1:400, Molecular Probes, #A-21203 and #A-11078). Cells were visualized with a 10×/0.30 or 20×/0.40 (dry lens) objective using an inverted microscope (DMI4000, Leica Microsystems) at room temperature in 1× PBS. The images were captured with a digital camera (DFC365 FX, Leica Microsystems) using LAS-AF software (Leica Microsystems). Confocal images were acquired with LSM 510 META (Zeiss) or Leica TCSSMD FLIM (Leica Microsystems) microscopes. The cells were visualized using Plan-Neofluar 20×/0.5 (dry) and Plan-Neofluar 40×/1.3 (oil) objectives using LSM 510 software (Zeiss) when the Zeiss microscope was used or HC PL APO 20×/0.7 (dry) and HCX PL APO 40× 1.25–0.75 (oil) objectives using LAS-AF (Leica Microsystems) when the Leica microscope was used. Following acquisition, the images were color corrected using the brightness, contrast, and color balance commands in Photoshop CS2 (Adobe Systems). This adjustment was applied to every pixel in each image.

### Flow cytometry analysis and CFSE proliferation assay

Nanog-GFP cells were analyzed using a Becton-Dickinson (Palo Alto) Accuri C6 flow cytometer and relative software. Differentiated cells were dissociated as single cells and resuspended once in PBS. Differentiated parental E14Tg2a ESCs were used as a negative control to subtract the autofluorescence.

To analyze proliferation of undifferentiated and differentiated wt and Hmga2 KO iPSCs using CFSE, adherent iPSC cultures were dissociated as single cells and washed three times with medium without FBS. Then, cells were incubated with CFSE (500 nM, Affymetrix eBioscience) at room temperature for 10 min in the dark followed by addition of cold excess of FBS and incubation for 5 minutes on ice. Stained cells were plated in ESC medium on gelatin-coated dishes (to reduce contamination of feeder cells) or induced to differentiate into EpiLCs or SFEBs. The CFSE content was evaluated by flow cytometry after 24 h for undifferentiated iPSCs, after 3 days for EpiLCs, and after 4 days for SFEBs. Proliferating cells show a lower CFSE staining than non-proliferating cells.

### Alkaline phosphatase staining

For alkaline phosphatase staining, wt HMGA2 KO iPSCs were cultured at clonal density (100 cells/10 cm^2^). The cells were fixed in 10 % cold Neutral Formalin Buffer (10 % formalin, 110 mM Na_2_HPO_4_, 30 mM NaH_2_PO_4_.H_2_O) for 15 minutes and then rinsed in distilled water for 15 minutes. The staining was obtained by incubation for 45 minutes at room temperature with the following staining solution: 0.1 M Tris-HCl, 0.01 % Naphthol AS MX-PO4 (Sigma), 0.4 % N,N-dimethylformamide (Sigma), and 0.06 % Red Violet LB salt (Sigma).

### Data analysis and statistics

The number of biological replicates (n) of each experiment is indicated in the Figure legends. The data values of each experiment are reported in Additional file 5.

Gene expression data obtained by qPCR were analyzed by the ∆Ct method and normalized to the expression levels of Gapdh using the means of 2^∆Ct^ of each experiment to calculate the standard error of the mean (SEM) or standard deviation (SD; as indicated in the legends) and to perform statistical analysis (when appropriate). For ChIP-qPCR experiments the results were analyzed using an average of Ct of no antibody and IgG as background. The 2^ΔCt^ of each sample was related to the 2^ΔCt^ of the input sample.

The percentage of total chromatin was calculated as 2^ΔCt^ × 10, where ΔCt = Ct(input) – Ct(immunoprecipitation).

*P* values were calculated with the Student *t*-test using a two-tailed test and paired samples.

## Additional files

Additional file 1: Generation and characterization of induced pluripotent stem cell (iPSC) clones derived from wildtype (wt) and Hmga2 knockout (KO) embryonic fibroblasts (MEFs). (A) MEFs derived from wt and Hmga2 KO mice were reprogrammed as described under Materials and Methods. The efficiency of reprogramming was evaluated after 21 days by staining for alkaline phosphatase activity. The number of positive colonies is reported in the graph as means of biological replicates ± SEM (n = 2). (B) Measurement of the reprogramming efficiency by counting Nanog-stained colonies at 21 days after reprogramming induction. The values are means of biological replicates ± SEM (n = 2). (C) expression of stemness markers in wt and Hmga2 KO iPSC clones by qPCR and western blot. The values are means of biological replicates ± SEM (n = 2). (TIF 625 kb) Additional file 2: (A) Different clones of wildtype (wt) and Hmga2 knockout (KO) induced pluripotent stem cells (iPSCs) were labeled with CFSE and the proliferation was evaluated based on the CFSE content by flow cytometry. Undifferentiated iPSCs were collected after 24 hours from CFSE labeling. Upper panels: histograms reporting the percentage of cells that underwent one or two divisions. Lower panels: representative FACS images of wt and Hmga2 KO iPSCs showing the different populations selected for CFSE content: M1, undivided cells; M2, cells after first division; M3, cells after second division. The values are means of independent experiments ± SEM (n = 3). (B) Analysis of the phenotype of Hmga2 KO iPSCs during neuron formation. wt and KO iPSCs were induced to differentiate and then collected at the indicated time points. The levels of stemness (Oct4, Nanog) and differentiation (Pax6) markers as well as of Otx2 were analyzed by qPCR. The values are means of biological replicates ± SEM (n = 2). (TIF 1628 kb) Additional file 3: (A) Specificity of HMGA2 antibody checked in chromatin immunoprecipitation (ChIP) assay performed on HMGA2 knockout (KO; induced pluripotent stem cells (iPSCs)) and knockdown (KD cells; epiblast-like stem cells). The values are means of biological replicates ± SEM (n = 3). **P* <0.05, ***P* <0.01. (B) Constitutive Otx2 binding to Hells enhancer was independent from Hmga2. ChIP-pPCR experiments demonstrated that the binding of Otx2 to the Hells enhancer in undifferentiated cells was similar in control cells and in Hmga2 KD (embryonic stem cells) or KO (iPSCs) cells. #1 and #2 are two independent Hmga2 KO iPSC clones. The values are means of biological replicates ± SD (n = 2). (TIF 242 kb) Additional file 4: Table S1. Sequences of the oligonucleotides used for q-PCR. (PDF 44 kb) Additional file 5: Supporting data. Data values of the experiments shown in the indicated Figures (shown in the sheet name) with the statistical analysis. (XLSX 176 kb)

## Abbreviations

ChIP: Chromatin immunoprecipitation
ESCs: Embryonic stem cells
EpiLCs: Epiblast-like stem cells
iPS: Induced pluripotent stem
KO: Knockout
KD: Knockdown
MEFs: Embryonic fibroblasts
q-PCR: Quantitative PCR
SFEBs: Serum-free embryoid bodies
TF: Transcription factor
wt: Wildtype
