# Supplementary figures and images for: Hmga2 is necessary for Otx2-dependent exit of embryonic stem cells from the pluripotent ground state
Source: BMC Biol. 2016 Mar 31;14:24. doi: 10.1186/s12915-016-0246-5 (PMC4818510; doi:10.1186/s12915-016-0246-5)

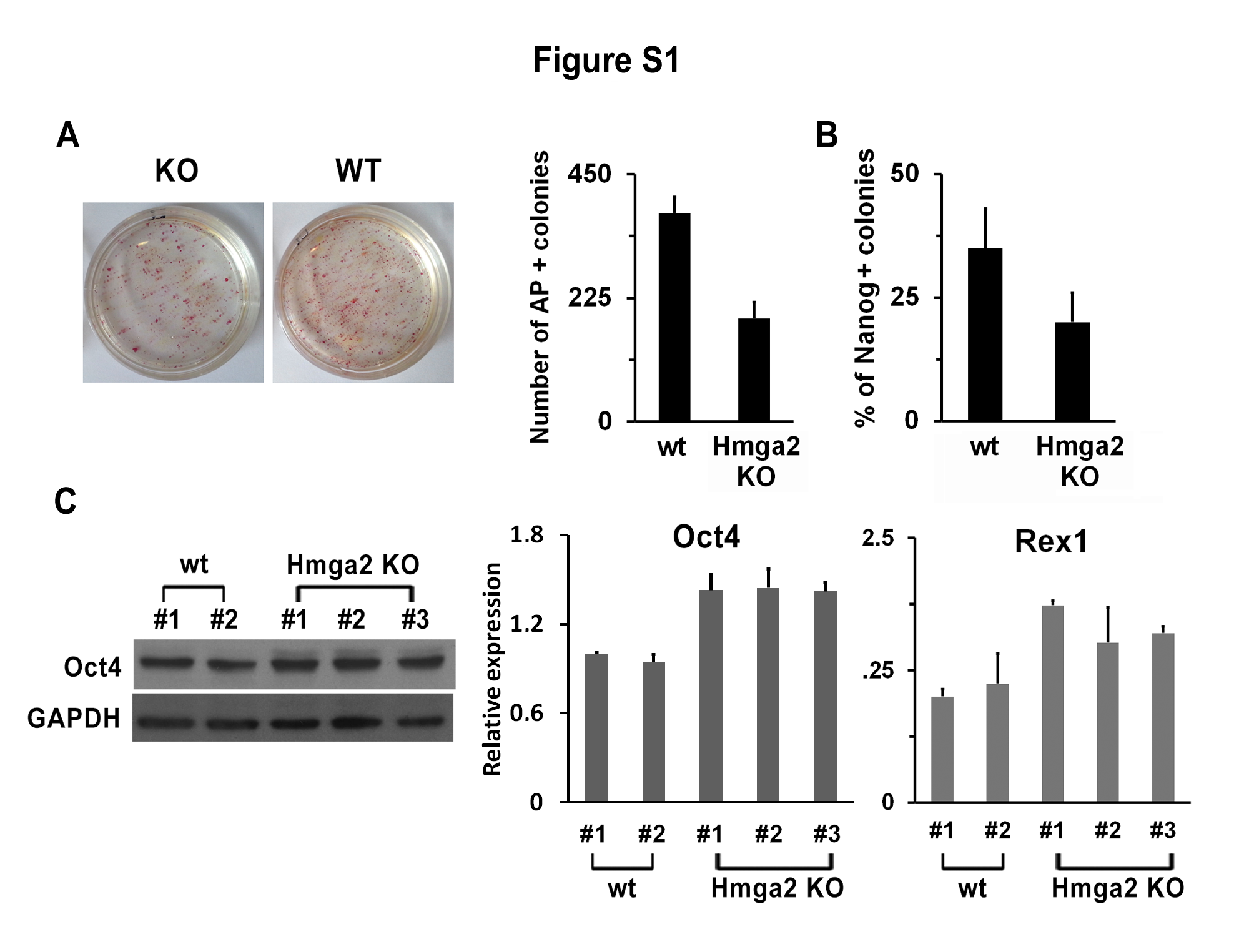

Supplement: Additional file 1: — Generation and characterization of induced pluripotent stem cell (iPSC) clones derived from wildtype (wt) and Hmga2 knockout (KO) embryonic fibroblasts (MEFs). (A) MEFs derived from wt and Hmga2 KO mice were reprogrammed as described under Materials and Methods. The efficiency of reprogramming was evaluated after 21 days by staining for alkaline phosphatase activity. The number of positive colonies is reported in the graph as means of biological replicates ± SEM (n = 2). (B) Measurement of the reprogramming efficiency by counting Nanog-stained colonies at 21 days after reprogramming induction. The values are means of biological replicates ± SEM (n = 2). (C) expression of stemness markers in wt and Hmga2 KO iPSC clones by qPCR and western blot. The values are means of biological replicates ± SEM (n = 2). (TIF 625 kb) [file 12915_2016_246_MOESM1_ESM.tif]

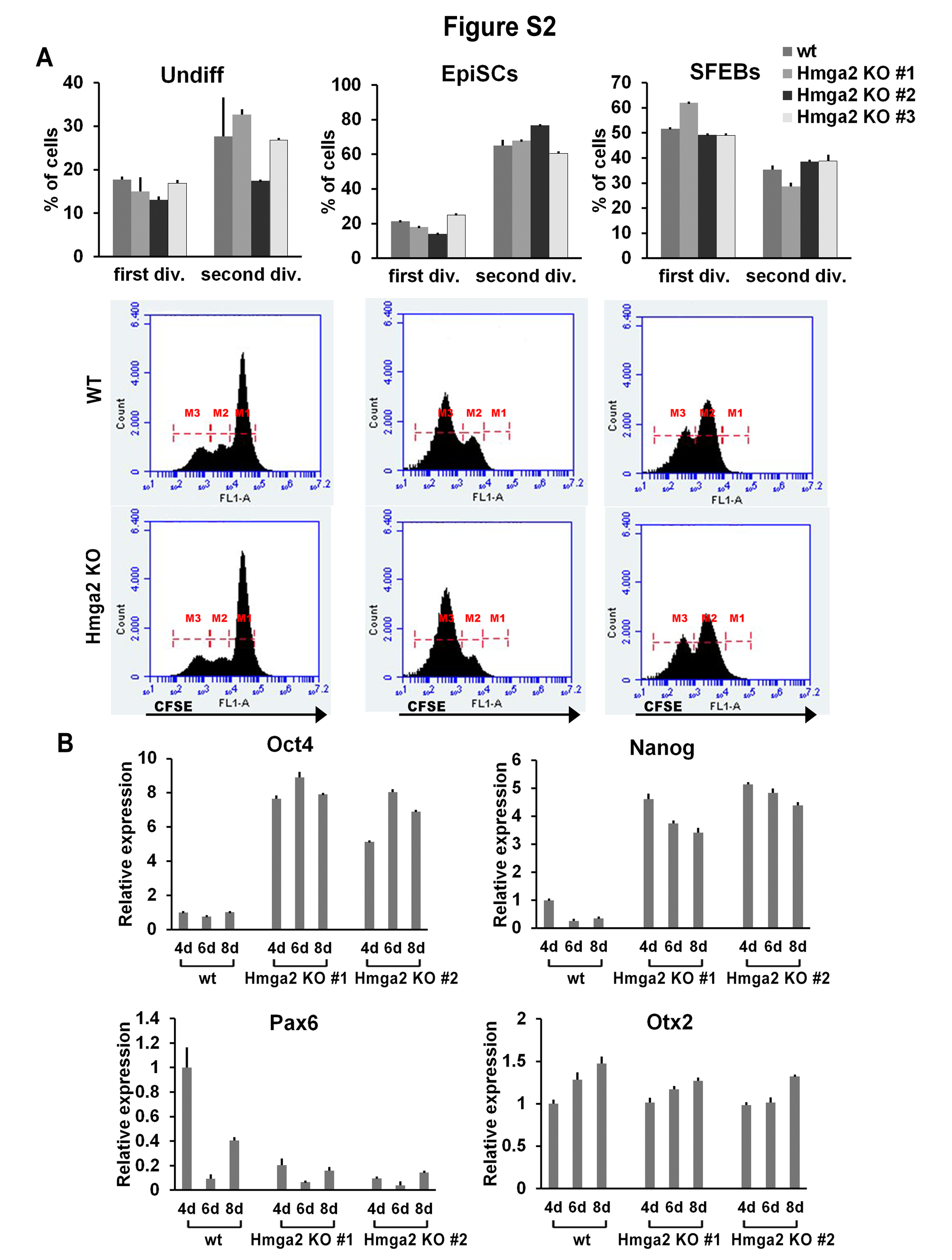

Supplement: Additional file 2: — (A) Different clones of wildtype (wt) and Hmga2 knockout (KO) induced pluripotent stem cells (iPSCs) were labeled with CFSE and the proliferation was evaluated based on the CFSE content by flow cytometry. Undifferentiated iPSCs were collected after 24 hours from CFSE labeling. Upper panels: histograms reporting the percentage of cells that underwent one or two divisions. Lower panels: representative FACS images of wt and Hmga2 KO iPSCs showing the different populations selected for CFSE content: M1, undivided cells; M2, cells after first division; M3, cells after second division. The values are means of independent experiments ± SEM (n = 3). (B) Analysis of the phenotype of Hmga2 KO iPSCs during neuron formation. wt and KO iPSCs were induced to differentiate and then collected at the indicated time points. The levels of stemness (Oct4, Nanog) and differentiation (Pax6) markers as well as of Otx2 were analyzed by qPCR. The values are means of biological replicates ± SEM (n = 2). (TIF 1628 kb) [file 12915_2016_246_MOESM2_ESM.tif]

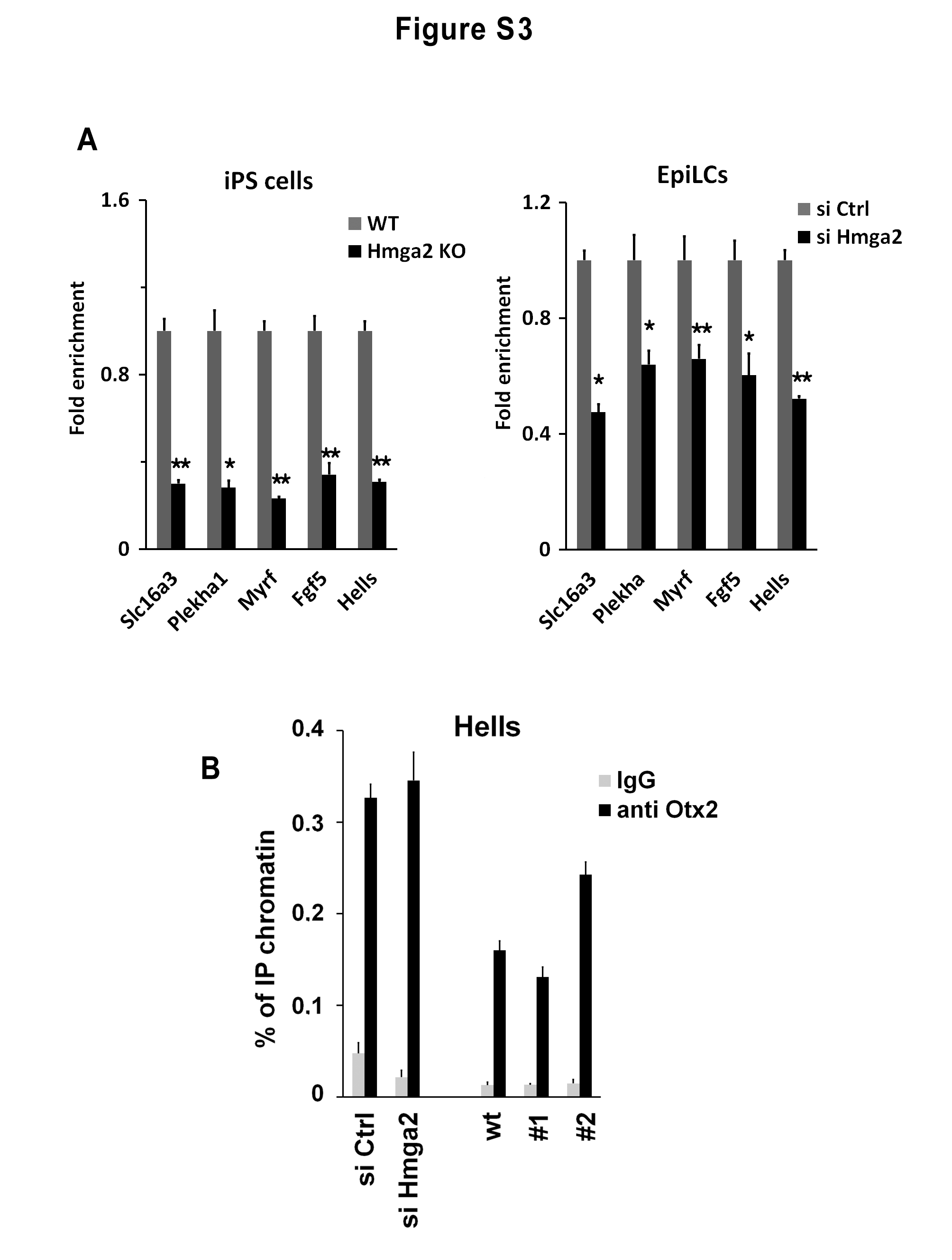

Supplement: Additional file 3: — (A) Specificity of HMGA2 antibody checked in chromatin immunoprecipitation (ChIP) assay performed on HMGA2 knockout (KO; induced pluripotent stem cells (iPSCs)) and knockdown (KD cells; epiblast-like stem cells). The values are means of biological replicates ± SEM (n = 3). *P <0.05, **P <0.01. (B) Constitutive Otx2 binding to Hells enhancer was independent from Hmga2. ChIP-pPCR experiments demonstrated that the binding of Otx2 to the Hells enhancer in undifferentiated cells was similar in control cells and in Hmga2 KD (embryonic stem cells) or KO (iPSCs) cells. #1 and #2 are two independent Hmga2 KO iPSC clones. The values are means of biological replicates ± SD (n = 2). (TIF 242 kb) [file 12915_2016_246_MOESM3_ESM.tif]
