# Supplementary material for: Hmga2 is necessary for Otx2-dependent exit of embryonic stem cells from the pluripotent ground state
Source: BMC Biol. 2016 Mar 31;14:24. doi: 10.1186/s12915-016-0246-5 (PMC4818510; doi:10.1186/s12915-016-0246-5)
Supplement: Additional file 4: Table S1. — Sequences of the oligonucleotides used for q-PCR. (PDF 44 kb) [file 12915_2016_246_MOESM4_ESM.pdf]

Table S1. Primers used for qPCR.

Name*	Application	Forward primer	Reverse primer
Dnmt3b	RT-qPCR	CCAAGGACACCAGGACGCGC	TCCGAGACCTGGTAGCCGGAA
Fgf5	RT-qPCR	TCCATGCAAGTGCCAAATTTACGGA	TTCTGTGGATCGCGGACGCA
Gapdh	RT-qPCR	GTATGACTCCACTCACGGCAAA	TTCCCATTCTCGGCCTTG
Gata4	RT-qPCR	GCCTGTATGTAATGCCTGCG	CCGAGCAGGAATTTGAAGAGG
Hells	RT-qPCR	GGCTGGCGGTAACAGAGTA	CGGCCTCGGACCTGAGTATT
Hmga2	RT-qPCR	AAAACGGCCAAGAGGCAGAC	ATGTCTCTTCAGTCTCCTGAGCA
Klf4	RT-qPCR	ACTCACACAGGCGAGAAACCTTAC	TCAGTTCATCGGAGCGGG
Myrf	RT-qPCR	GCCTTCAGCGTGGTGTCTAT	CTGGACCTGCCATCAGCATC
Nanog	RT-qPCR	TCAGAAGGGCTCAGCACCA	GCGTTCACCAGATAGCCCTG
Oct4	RT-qPCR	AACCTTCAGGAGATATGCAAATCG	TTCTCAATGCTAGTTCGCTTTCTCT
Oct6	RT-qPCR	GACGAGGATGCTCCCAGCT	AGCTTGATGCGTCGTTGCT
Otx2	RT-qPCR	TTCCGTCACTCCAAATCTACCCA	GCCGGACGGTCTCGATTCGCCTGGAGT
Plekha1	RT-qPCR	AACAGAGTGACATAATGATGAGGG	AGAGACTGCTTTAATCCAACTGTG
Rex1	RT-qPCR	GCAGTTTCTTCTTGGGATTTCAG	CTAATGCCCACAGCGAT
Slc16a3	RT-qPCR	GGCTGGCGGTAACAGAGTA	CGGCCTCGGACCTGAGTATT
Fgf5	ChIP-qPCR	TAAAAGCCACAGCAGGGACC	GACAAGAGGAGCCAGGTGAC
Hells	ChIP-qPCR	CACTTCCTCCCCCAGCTACT	TTCCGTTCTCCAGGGTTCCT
Hmga2	ChIP-qPCR	TGGTCCTTTTGCAGACTGGAT	GCACTGGTATTCACAACTGCC
Myrf	ChIP-qPCR	AGTGTCTGCCTTCTGCTTCC	GGGTGGCATGTTTGTCCCTT
neg ctrl	ChIP-qPCR	CAAGCCAGAGCCATGTGGCC	GGGGGGTGTGTGTGGCAAG
Oct6	ChIP-qPCR	CCGGGGCTCTGCTAGGATAA	CTACATCGAGAGGTTGCGCT
Plekha1	ChIP-qPCR	ATGGGTGGGTAGAGCCATCT	CAGCTCACTCACTGTCAGCA
Slc16a3	ChIP-qPCR	TGAGCAATGCAAATCCAGCG	ATCAGAGTGAGGGGCAAGGA

*The primer name is reported as indicated in the Figures.
